# Impact of Pharmacokinetic and Pharmacodynamic Properties of Monoclonal Antibodies in the Management of Psoriasis

**DOI:** 10.3390/pharmaceutics14030654

**Published:** 2022-03-16

**Authors:** Karine Rodríguez-Fernández, Víctor Mangas-Sanjuán, Matilde Merino-Sanjuán, Antonio Martorell-Calatayud, Almudena Mateu-Puchades, Mónica Climente-Martí, Elena Gras-Colomer

**Affiliations:** 1 Department of Pharmacy and Pharmaceutical Technology and Parasitology, University of Valencia, 46100 Valencia, Spain; karofer@alumni.uv.es (K.R.-F.); matilde.merino@uv.es (M.M.-S.); 2 Interuniversity Research Institute for Molecular Recognition and Technological Development, Polytechnic University of Valencia, 46100 Valencia, Spain; 3 Department of Dermatology, Hospital Manises, 46940 Valencia, Spain; antmarto@hotmail.com; 4 Department of Dermatology, University Hospital Doctor Peset, 46017 Valencia, Spain; amateupuchades@yahoo.es; 5 Foundation for the Promotion of Healthcare and Biomedical Research in the Valencian Community (FISABIO), 46020 Valencia, Spain; climente_mon@gva.es; 6 Department of Pharmacy, University Hospital Doctor Peset, 46017 Valencia, Spain; 7 Department of Pharmacy, Hospital Manises, 46940 Valencia, Spain; gras_ele@gva.es

**Keywords:** psoriasis, monoclonal antibodies, pharmacokinetics/pharmacodynamic models, therapeutic drug monitoring

## Abstract

The treatment of psoriasis has been revolutionized by the emergence of biological therapies. Monoclonal antibodies (mAb) generally have complex pharmacokinetic (PK) properties with nonlinear distribution and elimination. In recent years, several population pharmacokinetic/pharmacodynamic (PK/PD) models capable of describing different types of mAb have been published. This study aims to summarize the findings of a literature search about population PK/PD modeling and therapeutic drug monitoring (TDM) of mAb in psoriasis. A total of 22 articles corresponding to population PK/PD models of tumor necrosis factor (TNF)-α inhibitors (adalimumab and golimumab), interleukin (IL)-23 inhibitors (guselkumab, tildrakizumab, and risankizumab), IL-23/IL-12 inhibitor (ustekinumab), and IL-17 inhibitors (secukinumab, ixekizumab, and brodalumab) were collected. A summary of the clinical trials conducted so far in psoriasis was included, together with the current structural population PK and PD models. The most significant and clinical covariates were body weight (BW) and the presence of immunogenicity on clearance (CL). The lack of consensus on PK/PD relationships has prevented establishing an adequate dosage and, therefore, accentuates the need for TDM in psoriasis.

## 1. Introduction

Psoriasis is a chronic autoimmune and inflammatory skin disease associated with physical and psychological burdens characterized by erythematic plaques with adherent shiny scales [1]. The country-specific prevalence of psoriasis varies from 0.14% (95% uncertainty interval 0.05% to 0.40%) in east Asia to 1.99% (0.64% to 6.60%) in Australasia. Additionally, the prevalence is high in western Europe (1.92%, 1.07% to 3.46%), central Europe (1.83%, 0.62% to 5.32%), and North America (1.50%, 0.63% to 3.60%). Its age of onset shows a bimodal distribution, with peaks at 30–39 years and 60–69 years in men, and 10 years earlier in women [2]. The phenotypes of this disease are plaque psoriasis or psoriasis vulgaris, guttate psoriasis, inverse psoriasis, and erythrodermic psoriasis, which differ in terms of their clinical and morphological characteristics [3,4,5]. In addition, nail psoriasis is reported to affect more than half of the patients [6].

### 1.1. Pathophysiology of Psoriasis

**Figure 1:**
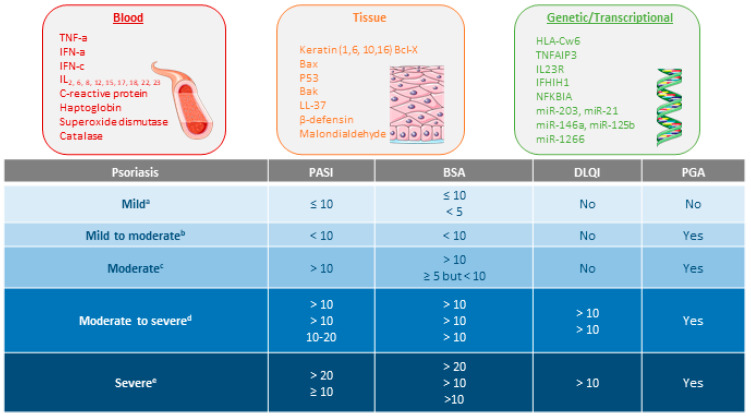
Types of biomarkers in psoriasis and psoriasis severity criteria according to several consensus guidelines or clinical associations. References supporting the consensus for ^a^ [20,21], ^b^ [22], ^c^ [21,22], ^d^ [20,22,23], and ^e^ [21,22,24].

A complex and not fully understood pathogenesis is exhibited in psoriasis. External factors can trigger an interaction between skin cells, pro-inflammatory immunocytes (i.e., tumor necrosis factor (TNF)-α and interferon (IFN)-α), and biologic signaling molecules in genetically predisposed individuals [7,8]. This interaction stimulates the myeloid dendritic cells (mDC) in the lymph nodes to release interleukin (IL)-12 and IL-23 to promote the cellular immune response of T helper lymphocytes (Th) type 1 (Th1), 17 (Th17), and 22 (Th22) T cells. Activated Th migrate to the skin guided by a gradient of chemokine and produce abundant psoriatic cytokines (i.e., IL-17, IFN-γ, TNF-α, and IL-22). The cytokine-mediated effects on keratinocytes influence typical psoriatic inflammation [9,10,11,12,13]. Molecular and genetic studies in specific psoriasis phenotypes have identified different inflammatory pathways that may coexist and evolve over time. The identification of the main inflammatory pathways through individual molecular descriptors represents a future step to guide personalized therapy [14]. In this sense, different classes of possible biomarkers have been explored in psoriasis (Figure 1), but further replication and validation are required [15,16,17].

### 1.2. Clinical Endpoints of Psoriasis

The severity of psoriasis will be determined by the extent of the disease, the location of the lesions, the degree of inflammation, and the impact on quality of life. According to the most important clinical guidelines (Figure 1), the evaluation of psoriasis severity and the levels of its treatment responses is generally based on the percentage of the total Body Surface Area (BSA) affected, Psoriasis Area Severity Index (PASI), Physician Global Assessment (PGA), and Dermatologic Life Quality Index (DLQI) [18,19].

## 2. Pharmacokinetic/Pharmacodynamic Properties of Monoclonal Antibodies in Psoriasis

Despite the increasing number of therapeutic monoclonal antibodies (mAb) on the market and in the drug development process for psoriasis treatment, the pharmacokinetic (PK) and pharmacodynamic (PD) properties of these molecules are more specific. In this regard, non-linear mixed-effects modeling allows for the accurate quantification of the central tendency and the different sources of the variability of mAb by considering data from all individuals simultaneously. The aims of this review are (i) to describe the main factors involved in the management of psoriasis disease with biological therapy, and (ii) to provide insights into the role of therapeutic drug monitoring (TDM) through population PK and PK/PD modeling strategies in the mAb treatment of patients with psoriasis.

### 2.1. Pharmacokinetic Properties

Monoclonal antibodies are heterodimeric glycoprotein macromolecules of type-G immunoglobulin recognizing a single epitope on a target antigen in a bivalent manner [25]. They are produced and engineered by hybridoma technology, developed for the first time by Köhler and Milstein in 1975 [26]. Due to their molecular size and their three-dimensional conformation, the PK and PD properties of mAbs are considerably different compared with those related to small-molecule drugs (SDM) [27].

The low permeability and high degradation of mAbs throughout the gastrointestinal tract lead to intravenous, subcutaneous, or intramuscular administration [28], and no significant improvement has been published to overcome the limitations of the oral administration of mAbs. Consequently, the most frequent routes of the administration of mAb in psoriasis follow intravenous (IV) or subcutaneous (SC) injections [29]. Regarding the distribution and tissue infiltration, mAbs can easily move from the SC space most probably via diffusion and/or convection through lymphatic capillaries, and they can be able to reach the intracellular space of targets beyond systemic circulation by pinocytosis or by receptor-mediated endocytosis [30].

The large size and physicochemical properties (charge and hydrophobicity) explain the distribution of mAbs mainly in the vascular and interstitial fluids. Usually, tissue distribution represents 5 to 15% of the total amount of mAb, and distribution into the brain is quite restricted (0.1%) [31]. A significant fraction of mAb in the body may be found if mAb-tissue target binding occurs with high affinity. Therefore, large apparent volumes of distribution in steady state (Vss) could be estimated for mAbs. In cases where the binding capacity of tissue is limited, nonlinear distribution is more probable and Vss decreases in a dose or concentration-dependent manner [32].

In regard to mAbs metabolism and excretion, the impacts of the renal and biliary pathways are insignificant [33]. Due to the null role of enzymatic processes related to the metabolism and excretion of mAb, the interaction with other substrates of these enzymes is negligible [34,35,36,37]. mAbs exhibit specific and non-specific types of binding, depending on the fragment of the antibody. The first one occurs when the antigen-binding fragment (Fab) attaches to the target antigen. The second one appears after the fragment crystallizable (Fc) region binds to cell surface receptors, such as the Fcγ receptor (FcγR) on the immune effector cells and the neonatal Fc receptor (FcRn) on different cell types, as well as components of the complement system (i.e., complement C1q) [25]. For such reasons, mAb distribution can be directly influenced by the density and expression of the target antigen. The two parallel metabolic pathways, i.e., specific and non-specific. are involved in mAb disposition, and their impact changes over time based on the available free mAb in the plasma and the dose administered. Metabolism through the reticuloendothelial system via pinocytosis/proteolysis represents the linear and non-specific clearance, which may be relevant at certain dose levels due to the larger endothelial surface area in the gut, muscle, and skin [38]. The specific pathway is initiated after the internalization of the receptor–drug complex, which allows the drug to enter the cell and then be inactivated by cytoplasmic endosomes. However, FcRn can bind IgG and mAbs at the acidic pH conditions of the lysosome, escape from proteolysis, and be directed back to the cell membrane [39,40,41]. Both pathways have been mechanistically described in population PK models through a target-mediated drug disposition (TMDD) approach and its quasi-equilibrium or rapid binding approximations, quasi-steady-state approximation, and even simpler Michaelis–Menten kinetics.

One more key aspect in the PK of mAb is the rescue from lysosomal degradation by binding to FcRn in endothelial cells, which is crucial for the long half-life and low clearance rate reported for most therapeutic mAbs [42,43]. These mechanisms result in clearance values of mAbs for psoriasis that range from 90 to 560 mL/day, leading to half-lives between 11 and 30 days. Several covariates have been identified in PK studies to partially explain inter-individual differences in mAb exposure, such as FcRn and FcγR gene expression, genetic polymorphism, target properties, and covariates associated with increased clearance, such as the generation of antidrug antibodies (ADA), low serum albumin and high serum C-reactive protein levels (CRP), gender, and high body weight (BW) [27,30].

### 2.2. Pharmacodynamic Properties

**Figure 2:**
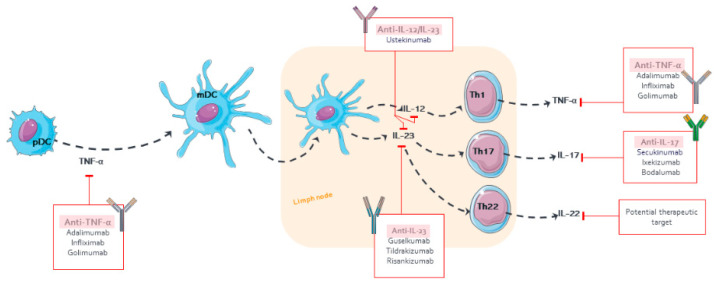
Pharmacological targets of monoclonal antibodies in psoriasis.

The mAbs for treating psoriasis are designed to block either the specific receptors or soluble mediators of the main pathways in the progress and chronicity of psoriasis, including TNF-α, IL-12/23, and IL-17 [11] (Figure 2). The PD effect of mAb is delayed to the time course of its plasma concentrations, which has been described using PK/PD models, such as indirect responses and transduction models, in order to describe the exposure–response (E–R) relationship [44,45]. The following parameters are mostly determined: kin, formation rate of psoriatic skin lesions; kout, remission rate of psoriatic skin lesions; Emax, maximum mAb effect; and EC_50_ or IC_50_, serum mAb concentration causing 50% of the maximum effect.

Compared with SDM, mAbs offer therapeutic exclusivity, a higher safety profile, and an increase in clinical efficacy [46,47]. The relative change in the PASI of patients receiving novel mAbs (guselkumab and brodalumab) has reached 90–100% PASI reduction, which has led to an adjustment of the primary endpoint of PASI75 to PASI90 or PASI100 in clinical trials [48] (Table 1).

## 3. Monoclonal Antibody Approved for Psoriasis

The selection of the optimal treatment for psoriasis depends on the severity of the disease [67]. Mild or limited-extent psoriasis is managed by topical treatment, while the moderate to severe types usually require a combination of phototherapy and systemic therapies [68]. Biological agents, such as mAb, have been the most successful approach in the management of this disease in the last decade (Table 1).

The use of mAbs is indicated in psoriasis when (i) effective control of psoriasis is not achieved with oral and phototherapy treatments, (ii) in patients who have rapid regrowth (3 months or less) after suspending any treatment, (iii) when higher doses of conventional systemic drugs are required with the increased associated risk of adverse effects, (iv) in patients with comorbidities for which the use of systemic agents, such as methotrexate or cyclosporine, are contraindicated, (v) when a patient is unable to tolerate the traditional systemic therapy, or (vi) the patient is at high risk of toxicity with methotrexate, cyclosporine, acitretin, or phototherapy, even in the absence of analytical alterations [23].

## 4. Population Pharmacokinetic/Pharmacodynamic Models for Monoclonal Antibodies in Psoriasis

An English systematic literature search was performed in databases of the field of Health Sciences—Embase, MEDLINE (via PubMed), and Scopus—to identify population pharmacokinetic/pharmacodynamic (PK/PD) models of therapeutic mAbs for the treatment of psoriasis. The following request was used in PubMed: (“Population pharmacokinetic/pharmacodynamic modeling” [Title/Abstract]) OR (“NONMEM” [Title/Abstract]) OR (“Exposure–response relationships” [Title/Abstract]) AND (“Name of the mAb” [Title/Abstract]) AND (“Psoriasis” [Title/Abstract]). Furthermore, to reduce the number of articles not recovered by the study, a manual search for population models was made by reviewing the bibliographies of relevant journal articles. The eligible articles were human-subject studies published between 1 January 2000 and 1 January 2021. The presented models were obtained through nonlinear mixed-effects modeling with the NONMEM software.

Table 2 shows the studies included in the population pharmacokinetic/pharmacodynamic models. Table 3 and Table 4 summarize the PK and PD outcomes, respectively, from the published models. The mAbs for which a PK or PK/PD model has been developed and published in patients with psoriasis are shown below.

### 4.1. Adalimumab

The PK properties of adalimumab and the factors influencing the adalimumab exposure levels in patients with moderate to severe chronic plaque psoriasis from phase II (M02-528) [69] and phase III (REVEAL) [51] clinical trials (Table 2) were characterized by Mostafa et al. [70]. The final structural model was a one-compartment model with linear elimination (Table 3). As in previous studies [113], the mean adalimumab concentration was between 5.2 and 18.2 µg/mL at week 12 (M02-528 study) and weeks 16 and 33 (REVEAL study). Additionally, patients with a reduction in the PASI score of at least 75% (PASI75 responders) achieved mean adalimumab concentrations three-fold higher than those in non-responder patients. The final estimates of the apparent clearance (CL/F) and the apparent volume of distribution (V/F) were similar to those observed in a previous investigation of patients with rheumatoid arthritis treated with the same dose of adalimumab [114]. The study type and BW were selected as statistically significant covariates, accounting for 19% and 29% of the variability in adalimumab CL/F and V/F, respectively. The assessment of immunogenicity on adalimumab efficacy and safety did not identify any significant relationship between positive and negative ADA patients, although CL/F was two-fold higher for positive patients, resulting in lower adalimumab exposure levels.

### 4.2. Golimumab

A population PK model approach allowed describing the concentration–time profile of golimumab and identifying patient and disease factors affecting its PK properties [71]. The study was performed in patients with active psoriatic arthritis (PsA) from a phase III study (GO-REVEAL) [55] (Table 2). The final structural PK model was a one-compartment model with first-order absorption and elimination. The population estimates for golimumab were CL/F = 1.38 L/d, V/F = 24.9 L, and k_a_ = 0.908 day^−1^, with IIV of 37.6% and 37.9% for CL/F and V/F, respectively. ADA, CRP, and smoking status were identified as significant covariates for CL/F, and BW for CL/F and V/F. The covariates inclusion reduced approximately 10% of the IIV for CL/F and V/F.

### 4.3. Ustekinumab

A population PK modeling approach was developed for ustekinumab in patients with moderate to severe plaque psoriasis [74] using two phase III clinical trials (PHOENIX 1 and PHOENIX 2) [56,57] and in patients with active PsA [73] from a phase II clinical trial (NCT00267956) [72]. A one-compartment open model with first-order absorption and first-order elimination was selected as the structural PK model for ustekinumab. The PK of ustekinumab was comparable between patients with psoriasis [74], patients with PsA from phase II [73] and phase III [79] studies, and real-world patients [77]. The attempts to incorporate the IIV in the term of ka were successful in the model-building process for PsA patients in the phase II study, but not for patients with psoriasis from PHOENIX 1 and 2, probably because a full characterization of the absorption phase of ustekinumab was limited due to the sparse sampling scheme designed for phase III studies.

Several covariates were identified and quantified, including BW, diabetes, and positive immune response ADA to ustekinumab (contributed to more than 20% of the changes in CL/F and/or V/F of ustekinumab for psoriatic [74] and PsA [73] patients (Table 3)). In addition, BW was reported to be the only covariate associated with increased V/F [77]. Based on the clinical relevance approach of the covariates, only BW justified a dose adjustment regimen for patients with moderate to severe psoriasis and PsA.

Once the PK of ustekinumab was determined, Zhou et al. [75] investigated the relationship between serum concentration–time data with longitudinal measures of psoriasis clinical severity using PASI. The effect, which accounted for the inhibition of the production of a p-40 subunit of both IL-12 and IL-23, was described by a sigmoid function with an indirect response model. The higher CL/F estimates for partial responders and non-responders suggested a decrease in the ustekinumab exposure levels compared with responder patients. The median serum drug concentration causing 50% of the maximum inhibitory effect (IC_50_) in responders was 30-fold lower than that in partial responders. Therefore, this study demonstrated that, to reach comparable efficacy, partial responders may require higher doses of ustekinumab and/or more frequent administration. A large IIV for IC_50_ was estimated (283%), but none of the tested covariables showed any significant relationship (Table 4). Moreover, the distribution of random effects of IC_50_ indicated an asymmetric bimodal distribution, but the inclusion of a mixed model did not substantially improve the fit. The predicted PASI 75 response rate from 100 replicates of the trials supported the observed PASI75 % response rates observed for both ustekinumab dose levels at week 28 in PHOENIX 1 and PHOENIX 2.

The second E–R model was performed by Pan et al. using real-world data of patients from a clinical site network integrating 60 dermatology centers across the United Kingdom (BSTOP and PSORTD studies) [77]. In general terms, similar results were obtained in both models; for instance, a substantial IIV of the serum drug concentration causing 50% of the maximum effect (EC_50_) (148.3%) was not associated with any of the tested covariates. However, unlike Zhou et al. [75], the use of a mixture model to account for the bimodal distribution of EC_50_ significantly improved the model fitting. In the mixed model, two subpopulations were identified: responder patients (EC_50_ = 0.07 µg/mL) and non-responder patients (EC_50_ = 1.21 µg/mL) (Table 4). The model simulations suggested that dose escalation/interval reduction may improve the probability of response in partial-responder patients, but not in non-responders. On the other hand, Pan et al. [77] demonstrated the clinical relevance of ustekinumab through the concentration at 4 (C_trough_) weeks and change in PASI from the baseline as a guide to determine the clinical outcome at 6 months, which can be included in a Bayesian therapeutic drug monitoring (TDM) algorithm to aid individualized ustekinumab dosing.

### 4.4. Secukinumab

The PK properties of secukinumab were characterized using pooled results from six clinical trials: five phase I or II studies and one phase III study in patients with psoriasis (Table 2) [58,80,82,83,84]. The PK data were best described by a two-compartment PK model with first-order absorption for SC administration and with zero-order infusion for IV administration [81]. Secukinumab shows a long half-life and slow serum clearance (CL) (0.19 L/day). The estimated volume of distribution is low, with a central compartment volume of distribution (VC) of 3.61 L and a peripheral compartment volume of distribution (VP) of 2.87 L. An allometric relationship between BW and CL and the volume of distribution characterized the influence of BW on the PK disposition parameters of secukinumab (Table 3).

### 4.5. Ixekizumab

The PK of ixekizumab was described by a two-compartment model with first-order absorption and elimination by Tham et al. [87]. An IIV higher than 200% was observed in the maximum placebo effect (PLBM) and EC_50_, but the model’s performance improved with the inclusion of the PASI75 responder status at week 12 as a significant covariate on the EC_50_ parameter. The population EC_50_ values of ixekizumab stratified by PASI75 non-responder and responder status at week 12 were 1.46 and 0.97 µg/mL, respectively (Table 4). This fact proved the existence of distinct levels of sensitivity to ixekizumab in patients and the possibility that non-responder patients may potentially become responders if they receive doses that allow them to receive sufficient exposure levels. Weight-related demographics, such as screening weight, BSA, and body mass index, did not affect the PASI scores.

Chigutsa et al. [88] described the relationship between the ixekizumab concentrations and the efficacy response in terms of static Physician’s Global Assessment (sPGA) and PASI via an ordered categorical model and a separate logistic regression model, respectively. The drug effect was linked through an E_max_ model with ixekizumab serum through the concentration levels at week 12. The models were able to accurately identify the proportion of responders using both efficacy measures, with higher concentrations associated with higher response levels. Higher concentration ranges were attained with 80 mg every 2 weeks, [90] which was associated with higher response levels. Even though factors, such as an increase in BW, higher baseline CRP concentration and palmoplantar psoriasis involvement, and lower baseline disease state, could statistically influence the response, none of them were clinically relevant (Table 4).

### 4.6. Brodalumab

Population PK models have been reported for brodalumab in plaque psoriasis patients, incorporating a two-compartment model with a depot compartment for SC absorption and parallel linear and nonlinear (Michaelis-Menten) elimination pathways [90,91,92]. The final estimates of the parameters of ka, Vc, Vp, CL, and maximal velocity for nonlinear elimination (V_max_) were similar between the three investigations (Table 3). Notable differences were found in the parameter estimates of CL and inter-compartmental clearance (Q). In the model developed by Timmerman et al. [91], CL and Q were approximately 25% and 53% lower, respectively, compared to the analyses of Endres et al. and Salinger et al., based on phase I and II data. Minor differences in V_max_ were found (6.07 vs. 4.39–5.40 mg/d). A fixed Km parameter was assumed by Endres et al. [90] (0.02 mg/mL), which was also considered by Timmerman et al. [91]. The mean predicted maximum concentration (C_max_) and area under the plasma concentration–time curve (AUC) at steady-state were 20 μg/mL and 225 μg day/mL, respectively [91]. The total BW had a significant impact on the CL, V_C_, and Vmax parameters, and no other covariates were identified as significant.

The PK–PASI relationship was characterized by Salinger et al. [92] through the inclusion of a signal compartment with an indirect response model of psoriatic plaques, where the signal suppressed plaque formation. The estimated IC_50_ was 2.86 mg/mL (SE: 50%) and the endogenous psoriatic plaque formation rate constant was 0.862 PASI units/day (Table 4).

### 4.7. Guselkumab

A confirmatory population PK analysis was implemented using a one-compartment linear model with first-order absorption and linear elimination, with IIV on CL/F and V/F [95,97]. The final estimates of the parameters were comparable between the two analyses, but parameter k_a_ was four-fold higher for Hu et al. [94] (4.93 1/d) than that for Yao et al. [97] (1.11 1/d) (Table 3). Some PK differences were explained through BW, which resulted in 28% and 32% IIV of CL/F and V/F, respectively. The model-predicted median steady-state minimum C_trough_ and AUC_tau_ after 100 mg SC administration of guselkumab every 8 weeks in psoriasis patients with a BW ≥ 90 kg was approximately 34% and 29% lower, respectively than those in patients weighing <90 kg. At the same time, guselkumab exposure was slightly reduced in diabetic patients, who had 12% higher CL/F than nondiabetic patients. However, no dose adjustment was recommended based on the BW bands [97].

Longitudinal, joint, and landmark E–R modeling analyses for two ordered categorical endpoints (PASI and PGA) [95,96] were performed with data from patients of phase II and III guselkumab clinical trials (Table 2). The estimates of k_out_ and E_max_ were comparable between the joint models, whereas IC_50_ was two-fold higher in the first published joint model for guselkumab [94] (Table 4). All models supported the guselkumab 100 mg every 8 weeks regimen as a cost-effective dose for the treatment of moderate to severe psoriasis Additionally, an effect of BW on E–R, independent of PK, was identified by Hu et al. [96].

### 4.8. Tildrakizumab

The population PK of tildrakizumab was described by a one-compartment model with first-order absorption and elimination kinetics, and IIV variability on CL, V_C_, and k_a_. The database contained information from six clinical trials, including information from healthy volunteers and patients with moderate to severe psoriasis (Table 2). The estimate of CL (0.32 L/day) was low and limited V_C_ (10.8 L) was obtained (Table 3). The absorption and elimination half-lives were 1.5 days and 23.4 days, respectively, with an absorption lag time of 1.2 h. With the clinical regimen, the steady-state was achieved by 16 weeks. Healthy subjects showed 31% higher bioavailability than those with lower BW [99].

An E_max_ logistic-regression E–R model was used to describe the week-12 PASI responses with the average concentration (C_avg_) of tildrakizumab during weeks 1–12 as the exposure metric (Table 4). At week 12, E_max_ was estimated at 62.2, 37.9, and 14.6% of responders for PASI75, 90, and 100, respectively. Individuals with higher BW had a lower response rate to placebo compared with lighter subjects. An indirect response PK/PD model with drug suppression of plaque formation and the placebo-induced healing rate was also developed to describe the longitudinal PASI reduction over 72 weeks [104].

### 4.9. Risankizumab

A population PK model of risankizumab was established with data from numerous clinical trials including patients with moderate to severe plaque, pustular, and erythrodermic psoriasis [105,108] (Table 2). The PK behavior of risankizumab was described using a two-compartment model with linear absorption and disposition (Table 3). The risankizumab steady-state exposures (C_max_, AUC_tau_, and C_trough_) following the administration of 150 mg SC in Japanese patients with pustular or erythrodermic psoriasis were approximately 17% higher than those in non-Japanese patients with moderate to severe plaque psoriasis. The covariate analysis identified several covariates on CL, but only BW and ADA showed clinically relevant changes in exposure [105,108].

The E–R relationships between the model-estimated risankizumab C_avg_ and the observed percentage of subjects achieving PASI75, PASI90, PASI100, and sPGA_0/1_ were characterized through an E_max_ model [110,111] (Table 4). The estimated EC_50_ values to achieve PASI75, PASI90, PASI100, and sPGA_0/1_ responses at week 16 and week 52 were significantly lower than the estimated C_avg_ value over weeks 0–16 and weeks 40–52. The estimated probabilities for the PASI75, PASI90, and sPGA_0/1_ responses were comparable at weeks 16 and 52. The covariate analysis identified high-sensitivity C-reactive protein (hs-CRP) as a statistically significant covariate for risankizumab EC_50_. Asian race was a statistically significant covariate for risankizumab EC_50_ for the PASI100 response at week 16. The exposure–efficacy relationships in Japanese patients were consistent with the relationships for patients in global phase III trials. A plateau of efficacy at week 16 was predicted after the 150 mg SC regimen, which resulted in PASI90 and sPGA_0/1_ response probabilities higher than 75%.

## 5. Therapeutic Drug Monitoring of Monoclonal Antibodies in Psoriasis

Treatments for an immune-mediated inflammatory disease, such as psoriasis, have been enhanced with the development of biologics. However, some patients are not able to achieve an adequate clinical response to mAb-based therapy. Some patients present an insufficient response in the induction phase of the treatment, which is called primary non-response, or after initial clinical benefit, they lose the ability to respond, which is called secondary non-response [115,116]. The IIV of the clinical response to standard biologic doses in patients with psoriasis may be explained by differences in the amount of drug available at the target tissue, which in turn is induced by adherence, physiological and genetic mechanisms, and PK covariates, such as BW and drug immunogenicity [117,118]. Increasing evidence indicates that a way to explain all these concerns about mAb could be TDM.

The term TDM was defined in 1997 by Watson et al. as the measurement of a prescribed xenobiotic in serum or biological fluids at a single or multiple time points, with a view to influencing prescription and individualizing the dosage regimen to achieve maximal clinical efficacy and minimize adverse effects [119]. Distinction should be made between reactive and proactive TDM. Reactive TDM is performed in patients failing treatment in order to guide decision-making, whereas proactive TDM is performed in responding patients to optimize therapy and potentially prevent future flare-ups and loss–of–response [120]. The implementation of TDM is essential to define the optimal dose ranges for each patient for a given biologic in psoriasis. The TDM for biological agents in immune-mediated inflammatory diseases involves the measurement of drug levels and ADA. Dose increase, interval shortening, and/or the addition of an immunomodulator are proposed, with subsequent re-evaluation of the drug concentration until the therapeutic goals are achieved [41].

In the last decade, the data in favor of TDM in psoriasis are growing. Based on the distribution of a survey among dermatologists who participated in Belgian Dermatology Days 2019 and Skin Inflammation & Psoriasis International Network Congress 2019, Schots et al. [121] indicated that 70% of the total study cohort admitted the need for TDM, implying the necessity in the daily dermatology routine for active interaction about the accessibility, utility, and application of TDM assays. However, over the years, there has been much confusion about what exposure metrics are informative in patients with psoriasis. Most of the studies reported in the literature have measured drug levels, but very little information has been used to evaluate the relationship between the mAb levels and clinical response to treatment [122]. Therefore, the selection of TDM in mAb for psoriasis may be beneficial due to the large IIV observed in clinical trials, its chronic administration that leads to the appearance of time-dependent changes in PK or PD parameters, and the role of disease progression in the increase of clearance and decrease in the response over time.

The attempts to establish therapeutic ranges and the incidence of ADA of some mAbs employed for the treatment of psoriasis are shown in Table 5. Takahashi et al. [123] identified the infliximab C_trough_ for responder patients at 0.92 μg/mL. Recently, the NORwegian DRUg Monitoring study was published [124] to assess the efficacy of TDM in patients on infliximab treatment regarding the achievement of remission, as well as to maintain immune-mediated inflammatory disease control. Additionally, among patients with immune-mediated inflammatory diseases undergoing maintenance therapy with infliximab, proactive TDM was more effective than treatment without TDM in sustaining disease control without disease worsening [125]. For adalimumab, Menting et al. [113] defined a window based on C_trough_ from 3.51 to 7.00 μg/mL corresponding to the optimal clinical response. This window was confirmed by the Psoriasis Stratification to Optimize Relevant Therapy (PSORT) consortium in a large multicenter prospective study [122]. Other studies have shown how early measurement of the adalimumab C_trough_ levels could help to predict the possibilities of responses [122,126,127].

A pilot study estimated a negative correlation between PASI and the trough secukinumab concentrations during maintenance therapy, suggesting no clinically relevant relationship between C_trough_ and PASI. On the other hand, a minimal effective C_trough_ of 33.2 μm/mL for achieving PASI ≤ 2 was proposed based on receiver operating characteristic curve analysis [128]. Menting et al. [129] reported low and variable trough concentration levels of ustekinumab, which were not correlated with clinical response. However, the studies by Toro-Montecinos et al. [130] and Van Den Berghe et al. [131] found an inverse correlation between the absolute PASI score and ustekinumab serum concentrations measured at week six and week four, respectively. These contradictory results have not made it possible to reach a consensus for the ustekinumab concentration–response relationship. Nevertheless, it has been demonstrated how early serum ustekinumab levels post-injection monitoring contribute to timely identifying under-exposed patients who might benefit from treatment optimization [77,131,132]. E–R association data in psoriasis is limited for certolizumab pegol, brodalumab, ixekizumab [87,88,133], guselkumab [96], tildrakizumab [102], and risankizumab [134].

**Table 5:** Therapeutic Drug Monitoring endpoints for biological drugs in psoriasis.

Drug	Incidence of ADA, %	C_trough_, μm/mL (Response)	Therapeutic Range
Etanercept	0.0–18.3 [49,50,126,135,136,137]	NA	NA
Adalimumab	6.5–45.0 [51,123,126,138,139]	3.51 [113], 7.84 [123], 9.7 [138] (PASI75)	3.51–7.0 [113]
Infliximab	5.4–54.2 [52,53,123,140,141,142,143,144,145,146]	0.92 [123] (PASI75)	NA
		3.16 [146] (PASI50)	
Ustekinumab	3.5–6.0 [57,132,147,148,149,150,151]	NA	NA
Secukinumab	0.3–0.4 [58]	33.2 [128] (PASI ≤ 2)	NA
Ixekizumab	9.0–13.4 [152]	NA	NA

Abbreviations: ADA, antidrug antibodies; C_trough_, minimum trough concentration; NA, not available.

## 6. Discussion

The current evidence of mAb treatments for psoriasis poses a challenge for clinical teams in the selection of dosage schedules that guarantee maximum efficacy and the lowest risk of toxicity. Therefore, the development and evaluation of quantitative frameworks that allow characterizing the time course of these molecules in the body and their response, together with the factors that explain the variability in the observations, has led to a revolution in the management of these patients.

Most of the scientific evidence of PK/PD modeling for mAbs in psoriasis was found in ustekinumab and risankizumab, while for others, such as golimumab and secukinumab, the number of publications regarding population PK modeling was very low. From a PK perspective, the structural models of the majority of mAbs have been described with a two-compartment model, which allows considering (i) the initial rapid decline and (ii) the peripheral distribution of mAbs into low-perfused tissues. The selection of a one-compartment model seems to be a result of limited PK sampling that does not allow the rapid disposition phase to be identified. In order to adequately characterize the disposition of this type of molecule, intensive sampling is necessary for the first few hours/days after mAb administration to identify the bi-exponential decline.

The majority of population PK models of mAbs assume linear disposition (linear distribution and elimination), and only brodalumab has partially described the non-linear PK properties using parallel linear and non-linear pathways. In our opinion, the lack of a wide range of dose levels able to visualize the saturation and synthesis of the receptor over time may limit the implementation of more complex (TMDD) structural PK models, since most of the clinical trials were conducted at an efficacious and safe dosage level. However, the use of linear PK models impedes extrapolation analysis for evaluating and proposing alternative dose levels or special sub-groups of populations (pediatrics, elderly, etc.).

Among the main covariates in the PK parameters, it is worth highlighting the influence of weight on the CL and V parameters in most mAbs. This may be due to the influence of the FcRn expression levels and greater interstitial tissue, which facilitates the existence of differences between individuals due to weight. Other covariates, such as albumin (ustekinumab, tildrakizumab, and risankizumab) and age (tildrakizumab and risankizumab), are relevant for the design of clinical trials that allow explaining the differences in the disposition of mAbs. The role of ADA in the mAb PK levels could be controversial, since there is great variability concerning the measurement kits used in each laboratory, so the data from each center are not comparable. This issue can be solved by the adoption of unified criteria, such as the designation of a central laboratory where all samples can be processed or the establishment of a universal kit that should be used by most centers to be able to compare results and draw definitive conclusions. To overcome the unpredictable PK variability of therapeutic mAb, model-informed TDM in patients with inflammatory bowel disease receiving infliximab has been recently suggested [120,153]. The use of population-based analysis to characterize the main PK and covariate effects in a target population, together with the relevance of TDM, could improve the dose-selection process of clinicians and reduce the use of non-optimal dosing schedules in psoriatic patients. In this sense, it is highly recommended to establish a successful PK/PD relationship that helps to understand the level of exposure needed to achieve a concrete efficacy/safety threshold.

Most of the studies reported in this review have evaluated the PK properties of mAbs, but little evidence has been provided to establish a mathematical relationship between PK and continuous or categorial PD endpoints. For this reason, academia, the pharmaceutical industry, and regulatory agencies are encouraged to jointly work to achieve the implementation of model-informed dosing of biological therapies to improve clinical practice in psoriasis. To this end, dose and schedule selection in clinical trials should be conducted not only accounting for the overall distribution of PK, but also PD variability, in order to select dosing regimens with optimal benefit–risk balance for the majority of the population. However, solid exposure–response relationships are rare due to the small number of dose levels tested, very sparse sampling, and high and flat efficacy rates that make it difficult to identify a quantitative and longitudinal relationship.

Some mAbs exert their pharmacological action through direct target binding and neutralization, followed by a downstream signal blockade, as is the case of adalimumab and golimumab. However, other mAbs (cetuximab and trastuzumab) recruit additional immune molecules to conduct the pharmacological effect [154,155]. So far, indirect response models [75,77,87,92,95,96,104] have been proposed to account for the pharmacodynamic response of mAbs on IL biomarkers. These structures make it possible to fulfill the assumption of biomarker synthesis in the absence of drugs and satisfactorily link the mechanism of interaction between the mAb and IL. However, PD baseline levels and PD observations during the recovery phase are required to properly characterize the system and drug related parameters.

Treatments for an immune-mediated inflammatory disease, such as psoriasis, have been enhanced by the development of mAb-based therapy. However, some patients are not able to achieve an adequate clinical response. Some patients present an insufficient response in the induction phase of the treatment, which is called primary non-response, or after initial clinical benefit, they lose the ability to respond, which is called secondary non-response [115,116]. The IIV of the clinical response to standard biologic doses in patients with psoriasis may be explained by differences in the amount of drug available at the target tissue, which, in turn, is induced by adherence, physiological and genetic mechanisms, and other covariates, such as BW and drug immunogenicity [117,118]. Increasing evidence indicates that a way to solve all of these concerns is the use of TDM, which aims to individualize dosage regimens to achieve maximal clinical efficacy and minimize adverse effects. Currently, the psoriasis management guidelines do not include the recommendation to use TDM, as is the case for other pathologies, such as inflammatory bowel disease [156]. In this sense, the identification of predictable PD biomarkers in plasma may help to anticipate the identification of responder and non-responder patients in the clinical setting. There is very little evidence of using the interleukin levels to predict the PASI index, but more efforts are needed in this way to clarify the contribution of early biomarkers to clinical responses. At the same time, they become an easy measurement of a direct endpoint linked with the mechanism of action of the mAb. Therefore, the use of a mathematical framework able to characterize the relationship between the PK, biomarker, and PD outcome over time is highly encouraged, together with the implementation of TDM, since both become an essential tool to define the optimal dose ranges for each patient for a given mAb in psoriasis.

## 7. Conclusions

This review represents the first attempt to compile all of the available information on population PK/PD models of therapeutic mAbs approved for psoriasis disease, including the clinical and regulatory information of the clinical trials conducted, population PK and PD parameter estimates, and the impact of significant covariates, which are of high relevance in the management of patients with moderate to severe psoriasis by clinicians. The PK properties of mAbs were described using a two-compartment model with linear absorption and disposition when sufficient PK evidence was collected. The characterization of the PD outcome was performed using an indirect response model to account for the change in PASI over time. Body weight was identified as a significant covariate for most of the mAbs, and ADA and age were included also for golimumab, ustekinumab, brodalumab, and tildrakizumab. The role of TDM for dose schedule selection in special sub-groups of patients has been revealed, showing the importance of having an adequate structural description of the PK and PD properties of mAbs, but also identifying relevant covariates that might influence the mAbs’ exposure or response. Despite the limited experimental evidence regarding the exposure–response relationship, the C_trough_ levels were summarized for adalimumab, infliximab, and secukinumab, which contributed to improving the model-informed dose selection process. Prospective analyses are encouraged to mathematically characterize the clinical exposure–efficacy relationships that contribute to establishing clinically relevant exposure endpoints for TDM and early detection of non-responder patients with psoriasis. Therefore, merging population PK/PD modeling and TDM, as a clinical decision support tool that allows knowing and predicting the clinical response in patients with moderate to severe plaque psoriasis, could be a key element to guarantee the efficacy of treatments with mAbs in psoriasis.

## Figures and Tables

**Table 1:** List of biologics approved for psoriasis.

Drug	Structure	Mechanism of Action	Route	Dosing Regimen	PASI75 (%)	PASI90 (%)	PASI100 (%)	Side Effect
Etanercept	Fusion protein	TNF-α receptor binding	SC	25 mg BIW or 50 mg QW	W12: 49 [49,50]	W12: 21–22 [49,50]		Infections, malignancies, and heart failure
Adalimumab	Monoclonal antibody	TNF-α binding	SC	80 mg LD + 40 mg Q2W	W16: 71 [51]	W16: 37 [51]	W16: 14 [51]	URI, nasopharyngitis, sinusitis, non-melanoma skin cancer, and heart failure thromboembolic events
Infliximab	Monoclonal antibody	TNF-α binding	SC	5 mg/kg induction at W 0, 2 and 6, then Q8W	W10: 75.5 [52]–80 [53] W50: 54.5 [52]–61 [53]	W10: 57 [53] W50: 34.3 [52]–45 [53]		URI, headache, fatigue, and squamous cell or basal cell cancers
Certolizumab pegol	Pegylated antigen-binding fragment	TNF-α binding	SC	200 or 400 mg Q2W	W16: 76–83 [54] W48: 81–87 [54]	W16: 44–55 [54] W48: 60–62 [54]	W16: 13–19 [54] W48: 24–38 [54]	URI, nasopharyngitis, and cell carcinoma
Golimumab	Monoclonal antibody	TNF-α binding	SC	50 or 100 mg Q4W	W14: 58 [55] W24: 66 [55]	W14: 24 [55] W24: 32 [55]		Infections and cutaneous squamous cell carcinoma
Ustekinumab	Monoclonal antibody	IL-12 and IL-23 binding	SC	45 or 90 mg at W 0 & 4, then Q12W	W12: 66.4–75.7 [56,57]	W12: 36.7–50.9 [56,57]	W12: 12.5–50.9 [56,57]	URI, nasopharyngitis, headache, and arthralgia
Secukinumab	Monoclonal antibody	IL-17A binding	SC	300 mg Ws 0–4, then Q4W	W12: 81.6–77.1 [58]	W12: 54.2–59.2 [58] W52: 58 [59]	W12: 24.1–28.6 [58] W52: 39.2 [58]	Nasopharyngitis, headache, and diarrhea during induction
Ixekizumab	Monoclonal antibody	IL-17A binding	SC	160 mg W 0, 80 mg Q2W for 3 months, then Q4W	W12: 77.5 [60] W60: 83 [60]	W12: 64.6 [59] W12: 59.7 [60] W60: 73 [61]	W12: 33.6 [59] W12: 30.8 [60] W60: 55 [60]	URI, nasopharyngitis, and headache
Brodalumab	Monoclonal antibody	IL-17A binding	SC	210 mg Q1W for 3 Ws, then Q2W	W12: 83.3 [61]–86.3 [62] W52: 80 [62]	W12: 69 [63] –70.3 [61] W52: 73–75 [62]	W12: 36.7–44.4 [62] w52: 53–56 [62]	URI, nasopharyngitis, headache, and arthralgia
Guselkumab	Monoclonal antibody	IL-23 binding	SC	100 mg at W0 & 4, then Q8W	W16: 86.5 [64]–91.2 [63] W48: 87.8 [63]	W16: 70 [63]–73.3 [63] W48: 76.3 [63]	W16: 34.1 [64]–37.4 [63] W48: 47.4 [63]	URI and nasopharyngitis
Tildrakizumab	Monoclonal antibody	IL-23 binding	SC	100 or 200 mg at W0 & 4, then Q12W	W12: 61–66 [65]	W12: 35–39 [65]	W12: 12–42 [65]	Nasopharyngitis
Risankizumab	Monoclonal antibody	IL-23 binding	SC	150 mg at W0 & 4, then Q12W	W16:88.7 [66] W52:92.8 [66]	W16: 73.2 [66] W52: 85.6 [66]	W16: 47.2 [66] W52: 60 [66]	URI, nasopharyngitis, and headache

Abbreviations: DNA, deoxyribonucleic acid; PDE4, phosphodiesterase-4; NF-κB, nuclear factor kappa B; TNF-α, tumor necrosis factor-alpha; IL, interleukin; IV, intravenous; IM, intramuscular; OR, oral; SC, subcutaneous; D, dose; W, week; QW, every week; QD, every day; BID, twice a day; TID, three times a day; BIW, twice a week; Q2W, once every 2 weeks; Q4W, once every 4 weeks; Q8W, once every 8 weeks; Q12W, once every 12 weeks; URI, upper respiratory infection.

**Table 2:** Published pharmacokinetics clinical trials of monoclonal antibodies indicated for psoriasis.

Drug	Study (Phase)	Disease	Dose Regimens	Subjects (Samples)	PD Endpoint (Samples)
Adalimumab	M02-528 (II) [69]	Pso	40 mg Q2W/QW (SC) [70]	827 P [70]	PASI, PASI75 [70]
	REVEAL (III) [51]				
Golimumab	GO-REVEAL (III) [55]	PsA	50 or 100 Q4W (SC) [71]	337 P (2029) [71]	
Ustekinumab	NCT00267956 (II) [72]	PsA	90 mg, W 0–3, 12, and 16 (SC) [73]	130 P (1594) [73]	
	PHOENIX 1 (III) [56]	Pso	45 or 90 mg, W 0, 4, then Q12W (SC) [74,75,76,77]	1937 P (9938) [74]	
	PHOENIX 2 (III) [57]			1312 P [75]	PASI (11624) [75]
	PSUMMIT I (III) [76] PSUMMIT II (III) [78]			925 P (2837) [79]	PASI (3429), ACR (8561) [79]
	BSTOP/PSORTD [77]			491 P (797) [77]	PASI (1590) [77]
Secukinumab	Hueber et al. (I) [80]	Pso	25–300 mg, then Q4W (SC) [81]	1233 P [81]	Total IL-17 [81]
	Rich et al. (II) [82]				
	Reich et al. (II) [83]				
	Papp et al. (II) [84]		1–10 mg/kg, sD (IV) [81]		
	ERASURE & FIXTURE (III) [58]				
	JUNCTURE (III) [85]				
Ixekizumab	I1F-MC-RHAJ (II) [86]	Pso	10–150 mg, W 0, 2, 4, then Q4W (SC) [87]	115 P (651) [87]	PASI (2096), PASI75 [87]
	UNCOVER-1 (III) [59]				
	UNCOVER-2 (III) [60]		80 mg Q2W/Q4W (SC) [88]	2888 P (2097) [88]	PASI, PASI75, sPGA [88]
	UNCOVER-3 (III) [60]				
Brodalumab	NCT00867100 (I) [89]	Pso	7–700 mg, sD (SC, IV) [90,91]	57 HV, 25 P [92]	PASI [92]
	NCT01937260 (I)		140 or 210 mg, sD (SC) [91]		
	NCT00975637 (II) [93]		70–280 mg first D, W 1, 2, then Q2W/Q4W (SC) [90,91,92]	196 P (1526) [90]	
	AMAGINE-1 (III) [61]		140 and/or 210 mg, W 0, 1, 2, then Q2W (SC) [91]		
	AMAGINE-2 (III) [62]		140 and/or 210 mg, W 0, 1, 2, then Q2W/Q4W/Q8W (SC) [91]	622 P (7725) [91]	PASI (2220), PGA (2456) [94]
	AMAGINE-3 (III) [62]				
Guselkumab	X-PLORE (II) [95]	Pso	1.5 mg Q12W (SC) [94]	238 P (2014) [94]	PASI (17580), PGA (18986) [96]
			15 mg Q8W (SC) [94]		
	VOYAGE 1 (III) [63]		50 mg Q12W (SC) [94,95,96,97]	1459 P (13031) [96]	
			100 mg Q8W (SC) [94]		
	VOYAGE 2 (III) [64]		200 mg Q12W (SC) [94]	1454 P (13014) [97]	
Tildrakizumab	P05776 (I) [98]	Pso	0.1, 0.5, 3 and 10 mg/kg, sD (IV) [98]	31 HV (340) [99]	
			50 or 200 mg, sD (SC) [98,99]		
	P06306 (I) [100]		10 mg/kg, sD (IV) [100]	53 HV (648) [99]	
			50–400 mg, sD (SC) [99,100]		
	P009 (I) [101]		200 mg, sD (SC) [99,101]	19 P (309) [99,101]	
	P05495 (IIb) [102]		5–200 W 0, 4, then Q12W (SC) [99,102]	349 P (4679) [99,102]	
	reSURFACE 1 (III) [103]		100 or 200 W 0, 4, then Q12W (SC) [99,103,104]	763 P (6329) [99]	PASI75, 90 and 100 [104]
	reSURFACE 2 (III) [103]			883 P (5016) [99]	
Risankizumab	NCT02596217/M16-513 (I) [105]	Pso	18–1200 mg, sD (SC) [105]	1899 HV and P (13123) [105]	
	NCT01577550/1311.1 (I) [106]		0.01–5 mg/kg, sD (IV), 0.25–1 mg/kg, sD (SC) [105]		
	NCT02054481/1311.2 (II) [107]				
	NCT03000075 (II/III) [108]		18 mg sD (SC), 90 or 180 W 0, 4, 16 (SC) [105,109]	2095 P [110]	PASI75, 90 and 100, sPGA [110,111]
	NCT03022045 (III) [109]				
	UltIMMa-1 (III) [112]		75 or 150 mg W 0, 4, then Q12W (SC) [110]	1903 P [110]	
	UltIMMa-2 (III) [112]				
	NCT02672852/IMMhance (III) [105]		150 mg W 0, 4, then Q12W or 150 mg Q12W (SC) [105,108,109,112]	1732 P [111]	
	NCT02694523/IMMvent (III) [105]				

Abbreviations: Pso, psoriasis; PsA, psoriasis arthritis; SC, subcutaneous; IV, intravenous; sD, single dose; W, week; QW, every week; Q2W, once every 2 weeks; Q4W, once every 4 weeks; Q8W, once every 8 weeks; Q12W, once every 12 weeks; P, patients; HV, healthy volunteers; PASI, Psoriasis Area and Severity Index; PGA, static Physician’s Global Assessment.

**Table 3:** Population pharmacokinetic parameters of monoclonal antibodies in psoriasis.

mAb	Model	PK Parameters													Covariates (Parameters)
		k_a_, 1/d (%RSE)	k_a_ IIV, %CV (%RSE)	CL, L/d (%RSE)	CL IIV, %CV (%RSE)	V_C_, L (%RSE)	V_C_ IIV, %CV (%RSE)	Q, L/d (%RSE)	Q IIV % (%RSE)	V_P_, L (%RSE)	V_P_ IIV, %CV (%RSE)	K_m_, µg/mL (%RSE)	V_max_, mg/day (%RSE)	V_max_, IIV, %CV (%RSE)	
Adalimumab [70]	1-CMT, LE	0.625 (28.8)		0.586 (3.8)	62 (8.6)	11.4 (5.6)	43.6 (31.5)								BW and study (CL/F)
															BW and study (V/F)
Golimumab [71]	1-CMT, LE	0.908		1.38	37.6	24.9	37.9								BW, ADA, CRP, and smoking (CL/F)
															BW (V/F)
Ustekinumab [74,75]	1-CMT, LE	0.354 (16.2)	0 (fixed)	0.465 (2.0)	41.0 (3.0)	15.7 (2.0)	33.2 (3.9)								BW, DB, ADA, Alb, CrCL, ALK, and sex (CL/F)
															BW, DB, and race (V/F)
Ustekinumab [77]	1-CMT, LE	0.23 (16.1)		0.44 (6.7)	44.7 (10.3)	10.2 (8.2)	36.5 (28.9)								Cr and ADA (CL/F)
															BW (V/F)
Secukinumab [81]	2-CMT, LE	0.18 (3.6)	35	0.19 (1.9)	32	3.61 (2.6)	30	0.39 (4.6)		2.87 (1.9)	18				BW (CL)
															BW (V_C_)
Brodalumab [92]	2-CMT, LE & NLE	0.255 (10.2)	75.1 (27.8)	0.28		3.9 (5.1)	29 (12.8)	1.01		2.89		0.01 (fixed)	4.39 (7.4)	31.5 (6.9)	
Brodalumab [92]	2-CMT, LE & NLE	0.236 (0.64)	57.9 (14)	0.223 (0.62)	69.2 (13)	4.62 (0.90)	69.6 (19)	0.697 (13)	15 (fixed)	1.84 (0.61)	85.6 (17)	0.02 (fixed)	5.16 (2.0)	37.8 (12)	BW and age (CL)
															BW and age (V_C_)
															BW and age (V_max_)
Brodalumab [91]	2-CMT, LE & NLE	0.300 (2.8)	62.6	0.155 (0.20)	57.5	4.68 (0.99)	25.5	0.328 (5.34)	91	2.41 (3.08)	189	0.02 (fixed)	6.07 (0.53)	2-CMT, LE & NLE	BW (CL)
															BW(V_C_)
															BW(V_max_)
Guselkumab [94]	1-CMT, LE	4.93 (4.9)		0.567 (3.7)	30.7 (23.3)	14.3 (3.4)	22.9 (26.0)								
Guselkumab [97]	1-CMT, LE	1.11 (14.1)	129 (22.9)	0.516 (1.19)	35.6 (6.54)	13.5 (1.08)	28.0 (9.85)								BW and DB (CL/F)
															BW (V/F)
Tildrakizumab [99]	1-CMT, LE	0.458 (6.8)	68 (17)	0.297 (1.1)	29 (5.9)	10.7 (1.1)	21 (15)								Age, BW, Alb, sex, race, and ethnicity (CL)
															Age, BW, and sex (V_C_)
Risankizumab [105]	2-CMT, LE	0.229 (4.8)	63 (5.5)	0.243 (1.8)	24 (3.6)	4.86 (3.8)	34 (6.6)	0.656 (3.7)		4.25 (2.0)					BW, Alb, Cr, hs-CRP, and ADA ≥ 128 (CL)
Risankizumab [109]	2-CMT, LE	0.230 (4.8)	3.36	0.244 (1.8)	5.5	4.87 (3.8)	11.1	0.648 (3.7)		4.25 (2.0)					BW, Alb, Cr, and hs-CRP (CL)
															BW (V_C_)
															BW (V_P_)

Abbreviations: CMT, compartment; LE, linear elimination; NLE, nonlinear elimination; F, bioavailability; k_a_, absorption rate constant; CL, clearance; V_C_, central volume of distribution; Q, intercompartmental transfer clearance; V_P_, peripheral volume of distribution; K_m_, Michaelis–Menten constant; V_max_, velocity for nonlinear elimination; IIV, interindividual variability, RSE, relative standard error; BW, body weight; DB, diabetes; ADA, antidrug antibodies; CrCL, creatinine clearance; CRP, baseline C-reactive protein level; Alb, albumin; ALK, alkaline phosphatase; Cr, serum creatinine concentration; hs-CRP, high-sensitivity C-reactive protein.

**Table 4:** Population pharmacodynamic parameters of monoclonal antibodies in psoriasis.

mAb	Model	PD Parameters								Covariates (Parameters)
		k_in_, PASI units/d (%RSE)	k_in_ IIV, %CV (%RSE)	k_out_, 1/d (%RSE)	k_out_ IIV, %CV (%RSE)	E_max_ (%RSE)	γ	EC_50_ or IC_50_ µg/mL (%RSE)	EC_50_ or IC_50_ IIV, %CV (%RSE)	
Ustekinumab [75]	Indirect response	0.615 (2.5)	60 (6.1)	0.0313 (1.9)	54 (4.7)	0.929 (0.2)	0.606 (3.4)		283 (7.2)	HTA, MTX (IC_50_)
										Sex (k_in_)
										Smoking (k_out_)
Ustekinumab [77]		SM: 15.5 (4.4)	SM: 0.02 (6.9)	SM: 43.6 (7.3)	SM: 43.6 (7.3)	1 (fixed)	SM: 0.14 (15.0)	SM: 148.3 (9.5)		
		MM: 15.8 (4.2)	MM: 0.02 (7.3)	MM: 41.4 (7.6)	MM: 41.4 (7.6)		MM 1: 0.07 (17.3) MM 2: 1.21 (22.2)	MM: 42.7 (58.2)		
Ixekizumab [87]	Indirect response	0.89		0.0564		1 (fixed)	0.776 (13.5)	R: 0.97 (60.3) NR: 1.46 (35.1)	R: 1660 (22.8) NR: 581 (44.2)	PASI75-W12 (EC_50_)
Ixekizumab [88]	Logistic regression (sPGA)					5.27 (3.6)		0.184 (34)		CRP (EC_50_)
										BW, PP (E_max_)
	Logistic regression (PASI75, 90, and 100)					6.02 (4)/5.54 (5)/5.73 (11)		0.354 (6)/0.268 (4)/0.169 (8)		BW, PP, baseline PASI (E_max_)
Brodalumab [92]	Indirect response	0.862 (40.1)		0.06389		1 (fixed)		2.86 (49.7)	136 (19.8)	
Guselkumab [94]	Latent variable indirect response (joint model)			0.0212 (5.69)		6.24 (4.93)		0.066 (21.3)		
Guselkumab [96]				0.0212 (1.96)		5.35 (1.54)		0.038 (6.22)		BW (IC_50_, k_out_)
Tildrakizumab [104]	Logistic regression (PASI75, 90 and 100)					PASI75: 62.16		PASI75: 0.36		
						PASI90: 37.89		PASI90: 0.46		
						PASI100: 14.63		PASI100: 0.55		
	Indirect response					1		0.25 (4.8)	183	
Risankizumab [109]	Logistic regression (sPGA)					sPGA: 0.431 (31.6)		sPGA: 0.916 (1.90)		hs-CRP (EC_50_)
	Logistic regression (PASI75, 90, and 100)					PASI75: 0.939 (1.21)		PASI75: 0.203 (34.3)		
						PASI90: 0.812 (2.44)		PASI90: 0.812 (2.44)		
						PASI100: 0.642 (9.49)		PASI100: 0.642 (9.49)		
Risankizu-mab [109]	Logistic regression (sPGA)					sPGA: 0.916		sPGA: 0.431		
	Logistic regression (PASI75, 90, and 100)					PASI75: 0.939		PASI75: 0.203		
						PASI90: 0.812		PASI90: 0.441		
						PASI100: 0.642		PASI100: 2.36		

Abbreviations: k_in_, formation rate of psoriatic skin lesions; k_out_, remission rate of psoriatic skin lesion; E_max_, maximum drug effect; EC_50_ or IC_50_, serum drug concentration causing 50% of the maximum effect; γ, Hill’s coefficient; IIV, interindividual variability, RSE, relative standard error; sPGA: static Physician’s Global Assessment; PASI: Psoriasis Activity and Severity Index; SM, single model; MM, mixture model; R, responder patients; NR, non-responder patients; PASI75-W12, PASI75 responder status at the week 12 primary; HTA, hypertension; MTX, past methotrexate use; CRP, baseline C-reactive protein level; BW, body weight; PP, palmoplantar psoriasis; hs-CRP, high-sensitivity C-reactive protein.

## Data Availability

Not applicable.

## Abbreviations

ADA	Antidrug antibodies
Alb	Albumin
ALK	Alkaline phosphatase
AUC	Area under the plasma concentration–time curve
BID	Twice a day
BIW	Twice a week
BSA	Body Surface Area
BW	Body weight
C_avg_	Average concentration
C_max_	Maximum concentration
CMT	Compartment
CL	Clearance
CL/F	Apparent clearance
Cr	Creatinine
CrCL	Creatinine clearance
CRP	Baseline C-reactive protein level
C_trough_	Minimum trough concentration
D	Day
DB	Diabetes
DLQI	Dermatologic Life Quality Index
DNA	Deoxyribonucleic acid
EC_50_	Serum drug concentration causing 50% of the maximum effect
E_max_	Maximum drug effect
E–R	Exposure–response
Fc	Fragment crystallizable
FcγR	Fcγ receptor
FcRn	Neonatal Fc receptor
γ	Hill’s coefficient
hs-CRP	High-sensitivity C-reactive protein
HTA	Hypertension
IC_50_	Median serum drug concentration causing 50% of the maximum inhibitory effect
IFN	Interferon
IIV	Inter-individual variability
IL	Interleukin
IM	Intramuscular
IV	Intravenous
k_a_	Absorption rate constant
Km	Michaelis–Menten constant
k_in_	Formation rate of psoriatic skin lesions
k_out_	Remission rate of psoriatic skin lesion
LE	Linear elimination
mAb	Monoclonal antibody
mDC	Myeloid dendritic cell
MM	Mixture model
MTX	Past methotrexate use
NF- κB	Nuclear factor kappa B
NLE	Nonlinear elimination
NR	Non-responder patients
OR	Oral
PASI	Psoriasis Area Severity Index
PASI75-W12	PASI75 responder status at the week 12 primary
PGA	Physician Global Assessment
PDE4	Phosphodiesterase-4
PD	Pharmacodynamic
PK	Pharmacokinetics
PK/PD	Pharmacokinetics/pharmacodymamics
PLBM	Maximum placebo effect
PP	Palmoplantar psoriasis.
PsA	Psoriatic arthritis
Pso	Psoriasis
Q	Intercompartmental transfer clearance
QD	Every day
QW	Every week
Q2W	Once every 2 weeks
Q4W	Once every 4 weeks
Q8W	Once every 8 weeks
Q12W	Once every 12 weeks
R	Responder patients
RSE	Relative standard error
SC	Subcutaneous
SM	Single model
SMDs	Small-molecule drugs
sPGA	Static Physician Global Assessment
TMDD	Target mediated drug disposition
TDM	Therapeutic drug monitoring
TID	Three times a day
Th	T helper lymphocytes
TNF-α	Tumor necrosis factor alpha
URI	Upper respiratory infection
V_C_	Central volume of distribution
V/F	Apparent volume of distribution
V_max_	maximal velocity for nonlinear elimination
V_Pz_	Peripheral volume of distribution
W	Week
